# Correlation between Acoustic Emission Behaviour and Dynamics Model during Three-Stage Deformation Process of Soil Landslide

**DOI:** 10.3390/s21072373

**Published:** 2021-03-29

**Authors:** Lizheng Deng, Hongyong Yuan, Jianguo Chen, Zhanhui Sun, Ming Fu, Fei Wang, Shuan Yan, Kaiyuan Li, Miaomiao Yu, Tao Chen

**Affiliations:** 1 Institute of Public Safety Research, Department of Engineering Physics, Tsinghua University, Beijing 100084, China; dlz17@mails.tsinghua.edu.cn (L.D.); zhsun@tsinghua.edu.cn (Z.S.); yan-s17@mails.tsinghua.edu.cn (S.Y.); li-ky18@mails.tsinghua.edu.cn (K.L.); yu-mm19@mails.tsinghua.edu.cn (M.Y.); 2 Beijing Key Laboratory of City Integrated Emergency Response Science, Tsinghua University, Beijing 100084, China; 3 Hefei Institute for Public Safety Research, Tsinghua University, Hefei 320601, China; fuming@tsinghua-hf.edu.cn; 4 Shenzhen International Graduate School, Tsinghua University, Shenzhen 518055, China; feiwang@tsinghua.edu.cn

**Keywords:** slope stability, acoustic emission monitoring, kinematics model, mechanical analysis

## Abstract

Acoustic emission (AE) monitoring has become an optional technology to quantify slope deformation. However, there are still challenges in developing generic AE interpretation strategies. Dynamics and kinematics models are two physical methods for analysing slope stability, which appear to improve the interpretability of AE monitoring data. The aim of this study is to explore the change patterns and interrelations of dynamics, kinematics, and AE measurements using a model test and physical analysis, to further understand the development process of a progressive landslide. A model test is designed based on the kinematics model of landslide three-stage deformation. An equation between factor of safety (FoS) and thrust is proposed based on the mechanical model of a landslide test. There is a clear correspondence between the displacement and inverse velocity during the deformation-controlled process. Relationships are uncovered between the thrust and FoS as well as the thrust and acceleration. As a characteristic parameter of AE, ring down count (RDC) is able to quantify the deformation process of the soil slope. Moreover, acceleration and RDC can reflect the sudden change of the slope state and, hence, can be effective indicators for the early warning in a progressive landslide.

## 1. Introduction

Landslides cause more than 4300 deaths and an economic loss of about $19.8 billion annually [1,2,3,4]. The actual losses caused by landslides are underestimated, as these losses are sometimes attributed to the consequences of their triggers, such as rainstorms and earthquakes [5]. The magnitude of the losses caused by landslides are more severe than those caused by other natural hazards, and they continue to increase with climate change, population growth, and mountain development [6,7,8,9,10,11].

Continuous slope monitoring can obtain the information of landslide evolution characteristics, and, hence, can be used for an early warning and emergency response. Deformation is a crucial physical parameter for slope monitoring and displacement is usually measured for decision-making [12]. Monitoring methods of landslide displacement can be divided into surface and subsurface technologies [13]. Radar, remote sensing, and GNSS (i.e., global navigation satellite system) are commonly used as surface monitoring technologies, which are vulnerable to the disturbance of atmospheric conditions, severe topographic fluctuations, and vegetation cover, and usually have low temporal resolution as well [14,15,16,17,18,19]. Inclinometer and ShapeAccelArray (SAA) are commonly-used subsurface monitoring instruments. However, the practical measurement range of inclinometer is limited while SAA is very expensive. Both inclinometer and SAA are not suitable for wide and long-term application in the field [13,20,21,22,23].

Acoustic emission (AE) technology has become an acceptable approach for landslide monitoring [23,24,25,26,27,28]. When particulate materials deform, part of the released energy is converted to sound and AE refers to the high frequency proportion of the sound energy [29,30]. AE technology has the characteristics of high temporal resolution, strain sensitivity, and lower costs. In addition, AE can be an effective method to quantify the deformation behaviour of a slope [23,27,31,32,33]. The evidence obtained in previous research shows that the AE rate is proportional to sliding velocity [23,25,33]. However, most of the studies on monitoring landslides using AE technology are based on laboratory tests and in-situ monitoring without mechanical analysis [23,27,31,32,33]. Physical analysis and calculation are required based on a dynamics model to further understand the mechanism of the landslide, and, hence, to interpret the AE measurements more deeply. The relationship between monitoring data and internal failure state of soil slope can be further investigated to improve the confidence of slope stability evaluation.

Physical analysis methods of slope stability mainly include a kinematics and dynamics model. The kinematics model distinguishes the evolution stage and stability level of the landslide based on the slope deformation/movement characteristics to provide a basis for disaster prevention. The common parameters considered in a kinematics model are displacement, velocity, and acceleration of the sliding body [34,35]. As a famous kinematics model, the three-stage deformation law of landslide is in accordance with the characteristics of the displacement-time curve of many landslides [12,36]. Moreover, Saito [37] proposed a method for predicting the remaining time to slope failure based on a three-stage creep curve. Fukuzono [38,39,40] developed Saito’s method, and proposed a simplified graphical method. Then, Fukuzono’s method has been widely used because the evolution state and failure time of the landslide can be acquired according to the trend of inverse velocity [12,41,42,43,44]. The essence of slope failure is the disturbed balance between sliding force and resistance, and the change of applied force at shear surface can reflect landslide evolution [45]. The dynamics model for slope stability analysis is generally based on the limit equilibrium theory. The factor of safety (FoS) equation of the slope is typically established as the ratio of shear strength to a sliding force on the failure surface [15,46,47]. FoS changes with the development of failure surface and directly quantifies the slope stability [48]. Both displacement and FoS are of clear physical significance and can reflect the whole process of landslide evolution. Many previous studies use kinematics or the dynamics model to analyse slope stability [49,50,51,52]. However, kinematics and dynamics model are seldom used together, and the benefits may be reflected by comparative validation of the two models. Using the kinematics and dynamics model together to analyse slope stability may improve the understanding of landslide activity, and improve the interpretability of AE monitoring.

In this study, a landslide model test is designed to simulate the simplified three-stage kinematic process of progressive landslides using a programmable logic controller. Data are obtained using an AE monitoring system, as well as the thrust and displacement measuring equipment. The main novelty of this study are four-fold: (1) three velocity equations are used to automatically simulate the three-stage deformation process of the landslide, (2) a concise FoS equation is established and the linear equation between the reciprocal of FoS and the thrust is derived, (3) physical parameters show a similar change pattern and the stability analysis result generated by the dynamics and kinematics model is consistent, and (4) the validated synergistic relationship among the acceleration, FoS, and ring down count (RDC) is helpful to comprehensively evaluate slope stability.

In the following sections, a landslide model test and AE monitoring are first introduced. Then, physical analysis is explained. Finally, the change patterns and interrelations of dynamics, kinematics, and AE measurements are explored based on four comparative experiments.

## 2. Methods

### 2.1. Experimental Detail

**Figure 1:**
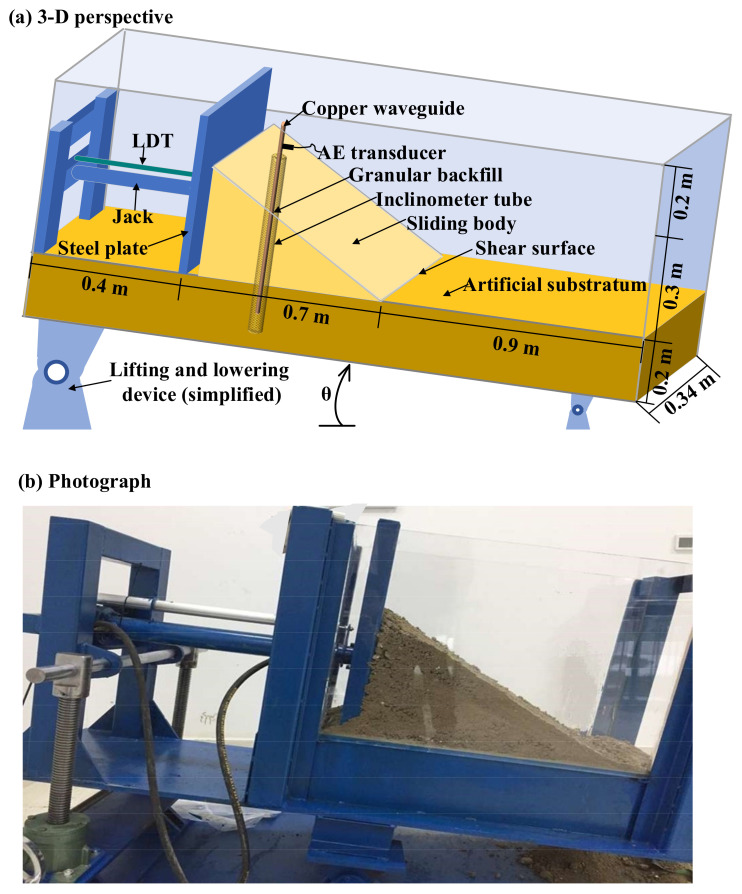
Landslide experiment system. For the 3-D perspective in (**a**), the loading equipment is indicated by the blue part, the artificial substratum is indicated by the brown part, and the sliding body is indicated by the yellow part. (**b**) is a photograph of the experiment system in a laboratory.

A landslide experiment system was built on the basis of the translational landslide model. The system was composed of a slope container (2 × 0.34 × 0.7 m), loading equipment, and several measuring instruments, as shown in Figure 1. The inclination angle of the container could be altered within a scope of 0–20°. Moreover, the physical parameters of the landslide test were measured, including thrust of the jack (using an internal hydraulic sensor), displacement of the sliding body (using a linear displacement sensor (LDT)), and AE parameters such us RDC. RDC is the number of times that the AE signal exceeds a pre-set voltage threshold [26].

Fine-grained sandy soil was found in some case studies on slope failure [53,54,55] and was selected as the slope material in this study. An effort was made to mix materials at the interface between artificial substratum and a sliding body to ensure slope material is continuous. The artificial substratum was compacted tighter than the sliding body while the sliding body was compacted continuously on the substratum. Hence, they formed as a whole at the start of each experiment. The properties of the sliding body were listed in Table 1. The internal friction angle of sandy soil was determined using the classic triaxial test [56,57,58]. The cohesion between the particles of sandy soil was neglected and, hence, a mechanical investigation was simplified.

**Figure 2:**
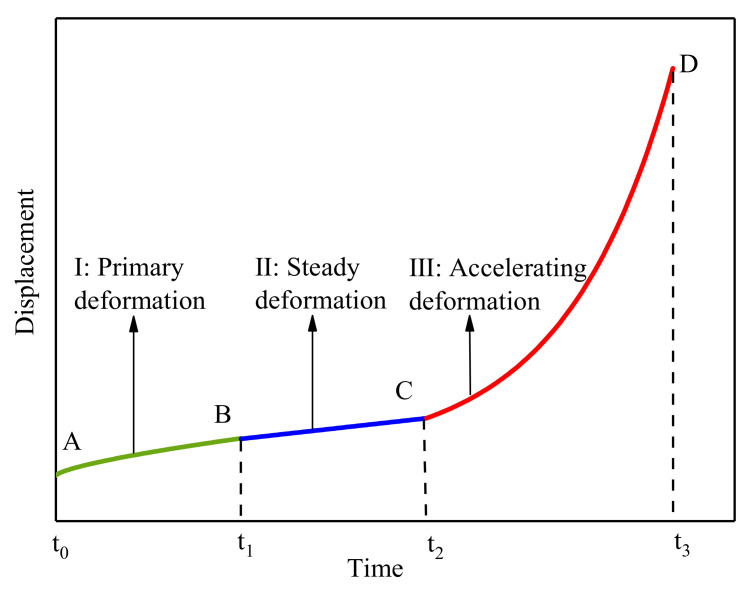
Three-stage deformation curve of a progressive landslide (after Saito [37]).

The experiment adopted the displacement-controlled method, and the loading process followed the kinematics model of a three-stage deformation law of progressive landslides. Figure 2 shows a typical three-stage deformation curve. Many landslides have experienced the process from slow deformation to accelerating movement [27,36,59], which may eventually cause rapid moving and destructive consequences [60,61].

The AE system includes a sensor, an amplifier, an acquisition board, and an analyser. The resonant AE sensor was used in this study, which had high sensitivity and could achieve a high signal-to-noise ratio. The contact surface of the AE sensor is made of piezoelectric ceramic material and the overall shielding casing can effectively reduce noise interference. When the collected AE signal enters the acquisition system, the modulated voltage signal is filtered and converted into a digital signal. The digital signal processing is then conducted to obtain the AE waveform and characteristic parameters. More details about the AE system and its working principle were explained in Deng et al. [61]. The dominant frequency range of the sensor was between 15 KHz and 70 KHz (i.e., lower sensitivity to a signal of other frequency bands), and the voltage threshold was set as 0.2 V during data acquisition. An active waveguide was used to avoid the high attenuation of AE signals propagating in soil and was stuck 6 cm into the artificial substratum. “Active” means the gap between the waveguide and the surrounding borehole is filled with gravel/sand and, hence, AE is mostly produced by the interaction of waveguide components [23,27,31]. When the host slope deforms, the backfill granular materials of active waveguide also deforms, and high levels of AE is generated and propagates along the metal waveguide. AE is produced by various physical mechanisms: bending of the waveguide, interaction between the backfill and the waveguide, and friction within the backfill materials.

Table 2 shows that tests (a–d), which were carried out to employ different inclination angles (5°, 10°, 15°, and 20°) of the container, to explore the performance of the sliding body under various stress states. The consistency between physical analysis and experimental results could be verified under multiple conditions. The tests in each inclined angle (5°, 10°, 15°, and 20°) were repeated three times and the provided results in Section 3 were the average measurements of three experiments.

### 2.2. Physical Analysis of a Landslide Model Test

The limit equilibrium method is typically used to check against ultimate limit state (ULS) failure. Either factors introduced to material parameters or a minimum FoS value against ULS failure is specified to explain uncertainties in the analysis. FoS describes an average shearing resistance along the shear surface that resists the applied shear force. Different stages of progressive landslide can be distinguished by the change of FoS [48]. Full mobilisation of shearing resistance and subsequent slope failure (or large displacement) would be expected when FoS reaches 1. In this study, FoS was calculated to deliver a physical model for analysing progressive landslide, and to provide a direct criterion to assess AE technology for identifying different slope behaviours.

The cohesion of sandy soil was neglected, and, hence, the shear strength was the friction resistance among particles. The shear strength of sandy soil was described in Equation (4) [62].

(4) $$\tau_{f}=\sigma \tan\varphi$$

where *τ_f_* represents the shear strength at the continuous interface between artificial substratum and sliding body, *σ* is the total normal stress on the shear plane, and *φ* denotes the friction angle of sandy soil.

**Figure 3:**
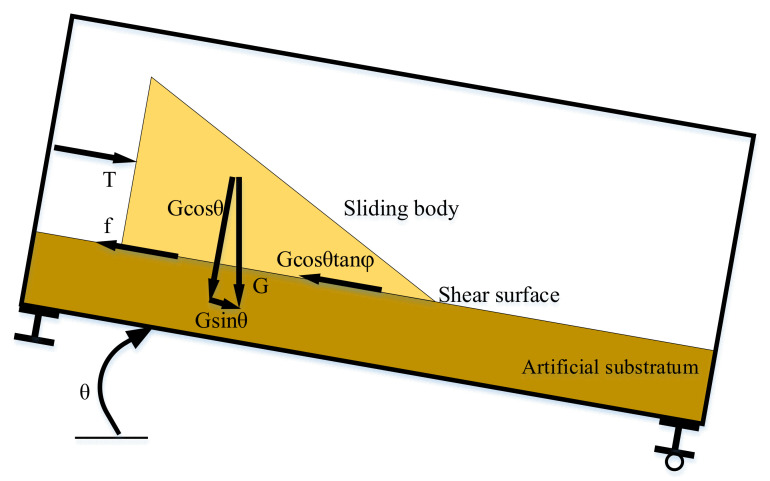
Diagram for mechanical analysis of the landslide test.

Figure 3 shows the mechanical analysis for the sliding body. Sliding force includes the component of gravity in parallel with the failure surface and the thrust of the jack. Resistance includes the friction from sandy soil and other resistance factors, such as the obstruction of the waveguide and the friction from both sides of the test container. FoS of the sliding body can be expressed in Equation (5) [63].

(5) $$FoS=\frac{G\cos\theta \tan\varphi +f}{G\sin\theta +T}$$

where *G* denotes the gravity of the sliding body, *θ* is the inclination angle of the test container (i.e., the angle of failure surface), *G*cos*θ* represents the normal stress, *φ* denotes the friction angle of sandy soil, and *φ* was measured to be 24°. *G*cos*θ*tan*φ* represents the friction at the bottom of the sliding body, *f* is the other resistance to the sliding body, *G*sin*θ* denotes the component of gravity in parallel with the failure surface, and *T* denotes the thrust of the jack. If FoS is greater than 1, the slope is in a safe and stable state. Otherwise, the slope is unstable and may undergo movement or even destruction. Of note, the stiffness of the waveguide (copper pipe) and rubber inclinometer seems higher than the unconfined pile of sandy soil, and it would introduce a challenge to estimate the other resistance *f*. However, the inclinometer gradually tilts down as the sliding body moves forward because the bottom of the inclinometer is not absolutely fixed, which is similar to an actual on-site monitoring situation [24,25]. The calculation of the other resistance f is explained in detail in Section 3.1.

## 3. Results and Analysis

In this section, the parameters of the dynamics model, kinematics model, and AE monitoring were analysed, and a quantitative relationship between several parameters was obtained. First, the average value of other resistance f was calculated using the thrust data from the steady deformation phase when the test container was at the horizontal level (θ = 0°). Additionally, f was used as a constant value for the calculation in subsequent experiments with different inclination angles. Second, a mathematical relationship between FoS and thrust was built. Then, the dynamics and kinematics analysis focused on the relationship between the displacement and inverse velocity, the thrust and FoS, as well as the thrust and acceleration. Third, the relationship between AE measurements and kinematics parameters was obtained. At last, comparative analysis was conducted using the acceleration of sliding body, slope FoS, and ring down count (RDC), which were selected as representative parameters for kinematics, dynamics, and Acoustic emission (AE), respectively.

### 3.1. Calculation of Other Resistance f and Simplification of the FoS Equation

AE monitoring instruments were installed for three-stage loading when the test container was at a horizontal level (θ = 0°). The initial phase of the steady deformation (i.e., 50–100 s in Table 2) was not stable enough and the thrust had a clear rising period of about 7 s. Hence, only the data of 57–100 s was used to calculate the other resistance *f*. The sliding body moved with a constant velocity and in a state of force balance. Equation (6) was proposed according to the mechanical equilibrium of the sliding body.

(6) f=T-Gtanφ

where *G* and tan *φ* are known. Thus, *f* can be obtained from *T*.

**Figure 4:**
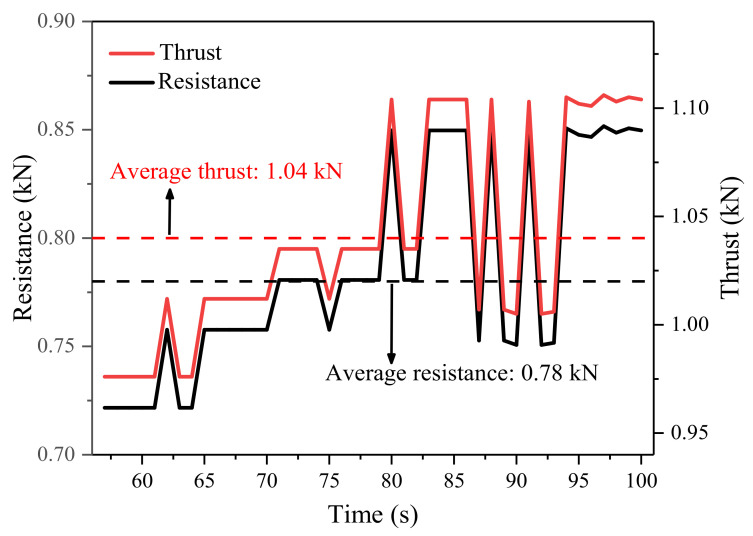
Time series of thrust and other resistance during a steady stage under a horizontal condition.

Figure 4 shows the data of *f* and *T* for the steady deformation phase. *T* increases slowly from 0.98 kN to 1.11 kN, with an average of 1.04 kN. Derived *f* increases gradually from 0.72 kN to 0.85 kN with an average of 0.78 kN. Both *T* and *f* increase slightly due to the dynamic change of other resistance factors and the measurement error of the force sensor. In order to eliminate the influence of these two uncertainties as much as possible and simplify the dynamics calculation, *f* was determined to be 0.78 kN. Only the inclination angle changed in the subsequent experiments. The movement process of the sliding body remained the same. To make the calculation concise, 0.78 kN was used as the substituted value for *f* in the subsequent analysis.

The loading thrust is parallel to the failure surface, and *G*, *θ*, *φ*, and *f* are known. Hence, the numerator and denominator of Equation (5) can be simplified using *a* and *b*, respectively. Equations (7)–(10) show the simplified process. Equation (9) shows FoS is inversely proportional to thrust, and Equation (10) determines the proportional relationship between the reciprocal of FoS and thrust *T*. Table 3 shows the calculated value of *a* and *b* by assigning 5°, 10°, 15°, and 20° to *θ* for tests 1 to 4, respectively.

(7) Suppose that       Gcosθtanφ+f=a

(8)         Gsinθ=b

(9) $$\mathrm{Thus}\;\;\;\;\;\;\;FoS=\frac{a}{b+T}$$

(10) $$\;\;\;\;\;\;\;\;\;\frac{1}{FoS}=\frac{T}{a}+\frac{b}{a}$$

**Figure 5:**
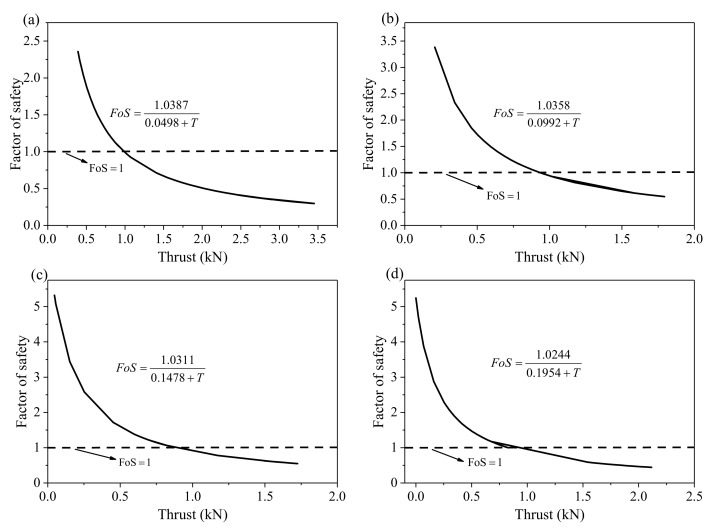
The factor of safety (FoS) is inversely proportional to the thrust. (**a**–**d**) show the inverse relationship between FoS and thrust for the four experiments.

**Figure 6:**
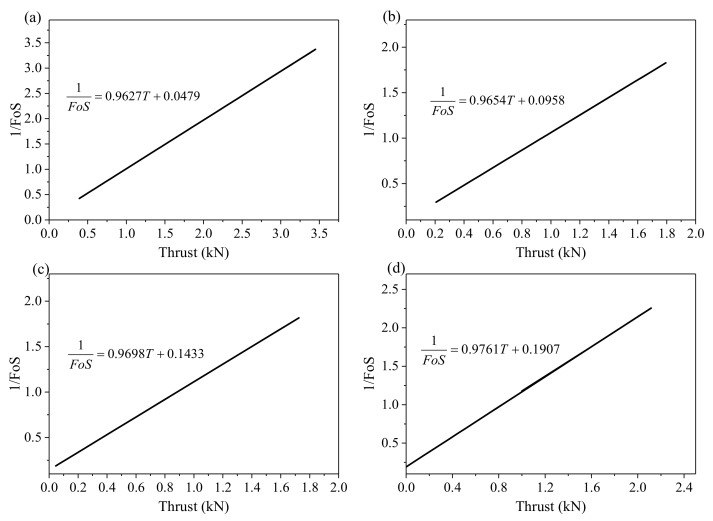
Reciprocal of the factor of safety (FoS) is proportional to the thrust. (**a**–**d**) show the proportional relationship between reciprocal of FoS and thrust for the four experiments.

Figure 5 and Figure 6 show the quantitative relationship between FoS and thrust determined by Equations (9) and (10) for each experiment. These curves clearly show that FoS is inversely proportional to the thrust and the reciprocal of FoS is proportional to the thrust. When FoS equals to 1, thrust can be determined from the curve and, hence, thrust can be used for identifying whether the slope is stable.

### 3.2. Analysis of Kinematics and Dynamic Parameters

#### 3.2.1. Displacement and Inverse Velocity

**Figure 7:**
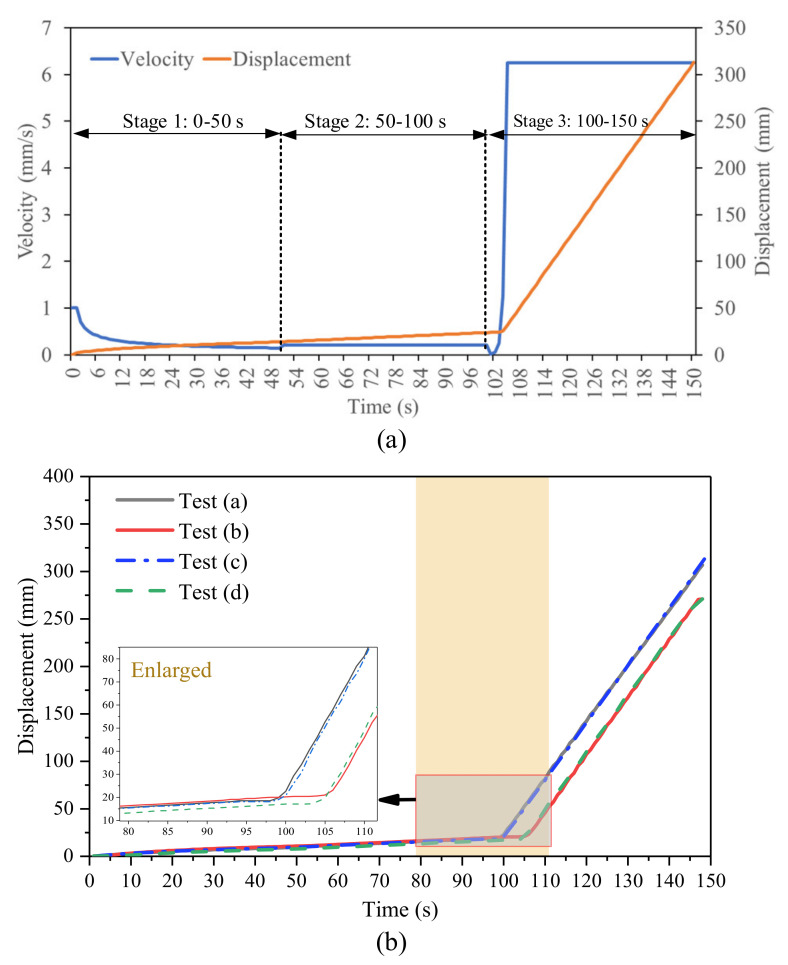
Landslide kinematics parameters of the model test. (**a**) This is expected displacement and velocity change with time based on the velocity-controlled method in Table 2. (**b**) This is measured displacement plotted against time for each test.

Figure 7a shows the expected displacement and velocity change with time based on the details of velocity control in Table 2. Figure 7b shows the measured displacement curve from four tests increasing with time and each test lasts for about 150 s. When the accelerating stage begins at about 100 s, the displacement curve rises sharply with the rapid deformation of the slope. The actual accelerating stage only lasts for a few seconds due to an exponential function of the designed velocity equation and limited capability of the loading device. Hence, the increasing velocity reaches the maximum value soon and the highest velocity remains stable at about 6 mm/s.

**Figure 8:**
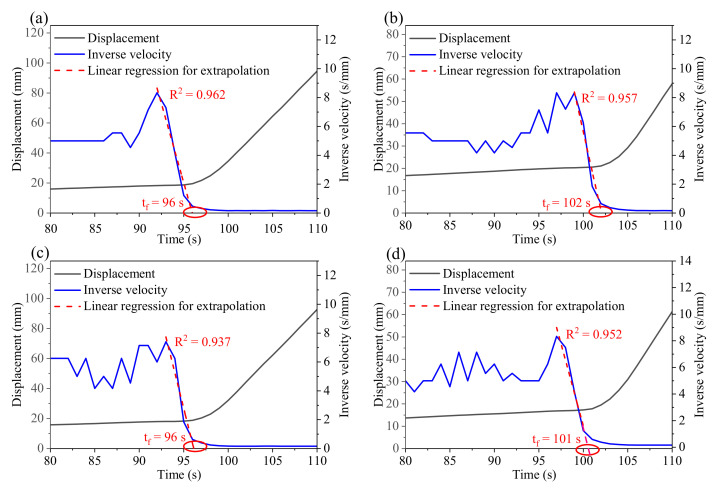
Displacement and inverse velocity plotted against time (80–110 s) in (**a**–**d**) for the four experiments. All data points are the average value of 1 s.

The derivatives of displacement, such as velocity and acceleration, are directly related to slope stability [34]. Moreover, the evolution state and failure time of a landslide can be judged according to the trend of inverse velocity. Considering Fukuzono’s method [40], the accelerating moment of the sliding body is taken as the starting point of failure prediction. When velocity gradually increases, the inverse velocity decreases, and the curve extrapolated from the inverse velocity eventually intersects with the time axis, which determines the failure time tf [38,39,40]. Figure 8 shows the calculated inverse velocity based on the displacement data of 80–110 s in Figure 7. The displacement curve increases exponentially and the extrapolation curve of inverse velocity intersects the time axis at about 100 s. Linear fitting is used for the accelerating stage of the inverse velocity curve, and R^2^ is near 0.95. When the inverse velocity reaches its lowest point, the velocity reaches its maximum value and remains constant at about 6 mm/s. In these four landslide experiments, failure time cannot be regarded as the occurrence of slope collapse. However, large acceleration leads to drastic changes in the motion state of the sliding body, which may cause serious damage. From the perspective of public safety, the prediction of failure time is crucial for preventing emergencies.

#### 3.2.2. FoS and Thrust

**Figure 9:**
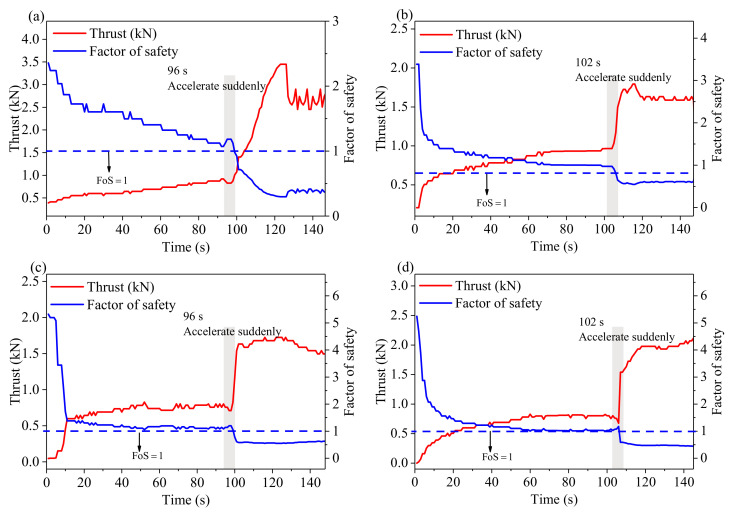
Thrust and factor of safety (FoS) plotted against time in (**a**–**d**) for the four experiments. All data points are the average value of 1 s.

The change of FoS with time can be calculated using Equation (10) and thrust data. Figure 9 shows FoS and thrust time series. The changes of thrust and FoS are different in four experiments, but reveal a consistent pattern of landslide evolution. In general, the thrust increases while the FoS decreases with time. They almost change synchronously when the accelerating stage appears at around 100 s. The thrust curve rises sharply while the FoS drops below 1, which indicates the slope enters an unstable state. The same conclusion can be drawn from the force analysis of the sliding body and Equation (5). Accelerating stage means the total sliding force is greater than the resistance. Hence, FoS is less than 1.

#### 3.2.3. Acceleration and Thrust

**Figure 10:**
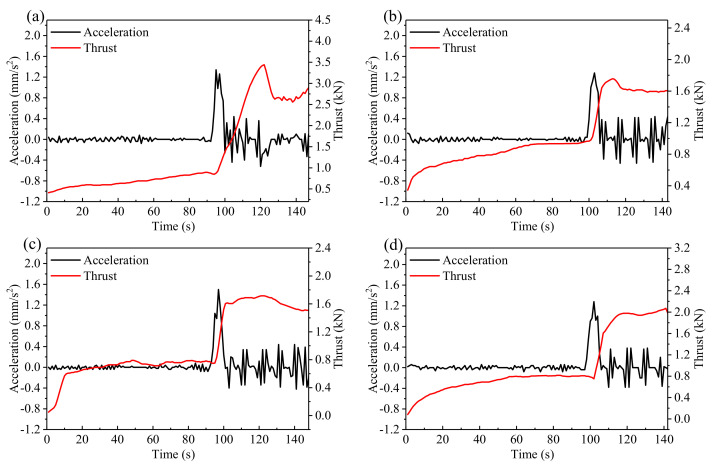
Acceleration and thrust plotted against time in (**a**–**d**) for the four experiments. All data points are the moving average value of 5 s.

Acceleration is the result of combined external forces, and it connects force with motion. In terms of three-stage deformation of the landslide, acceleration of the sliding body responds to slope destabilizing effects. A rapid increase of acceleration indicates slope failure may occur soon. Thus, detection and quantification of acceleration may provide early warning of slope failure [13,64,65,66]. Figure 10 shows the thrust and acceleration time series. Before 100 s, thrust increases slowly and acceleration oscillates slightly near 0. When the third stage begins at about 100 s, thrust increases sharply. Meanwhile, acceleration jumps and reaches the peak value (more than 1.2 mm/s^2^). Then, acceleration oscillates slightly and its amplitude does not exceed 0.5 mm/s^2^. The change of acceleration is prominent and closely related to landslide evolution. Therefore, acceleration may become a quantitative indicator for incipient slope failure and an early warning.

### 3.3. Analysis of AE and Kinematics Parameters

#### 3.3.1. RDC and Velocity

**Figure 11:**
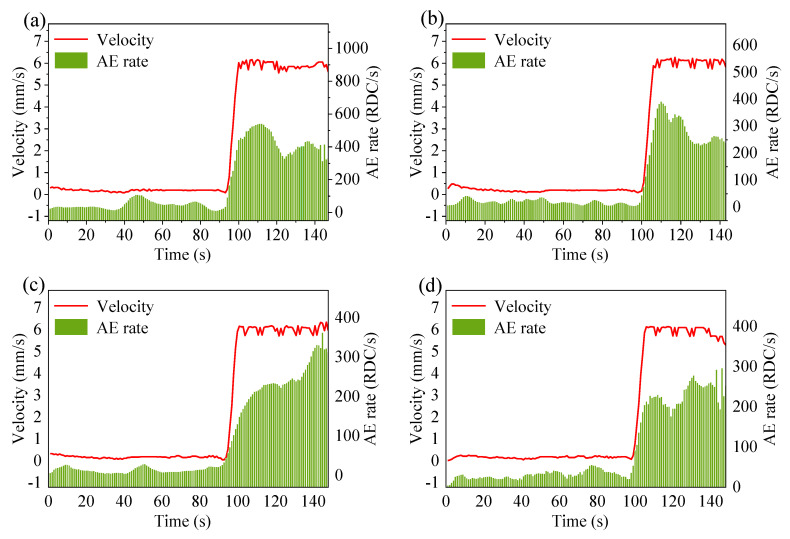
Velocity and ring down count (RDC) plotted against time in (**a**–**d**) for the four experiments. All experimental data points are the moving average value of 5 s.

RDC obtained by AE monitoring responds to the continuous deformation of the slope. The measured AE data were depicted with a smoother value of five-second moving average (FSMA) values. The FSMA values were determined by calculating the average of the AE rate (RDC per second) over the 2 s preceding and 3 s succeeding each measurement. The moving average processing provided a continuous time representation of the AE rate and improved the characterization performance of AE activity because this representation was more informative within a time period. The correlation between the TSMA AE rate and landslide velocity was expected to be more synchronous. Figure 11 shows the variation of velocity and RDC with time for each experiment. The trend of velocity and RDC is consistent. Both are relatively small and stable in the first two stages, but increase dramatically at about 100 s. The sharp increase of velocity leads to a stronger interaction between particles, with contact stress being released and force chain buckling, which then causes higher AE activity and increased RDC [67].

#### 3.3.2. Cumulative RDC and Displacement

**Figure 12:**
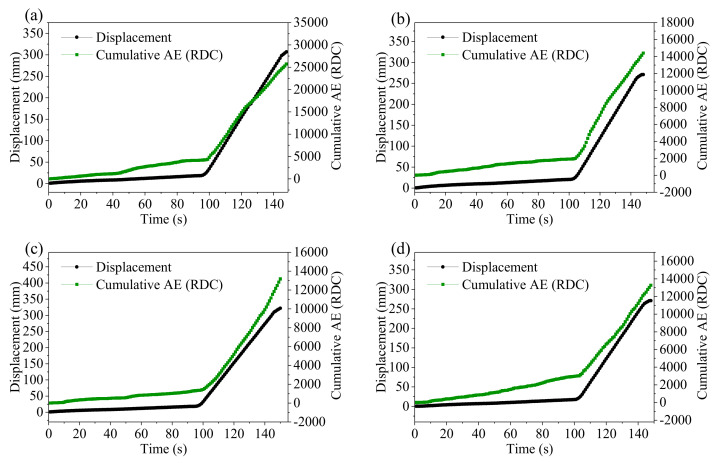
Displacement and cumulative ring down count (RDC) plotted against time in (**a**–**d**) for the four experiments. All data points are the moving average value of 5 s.

**Figure 13:**
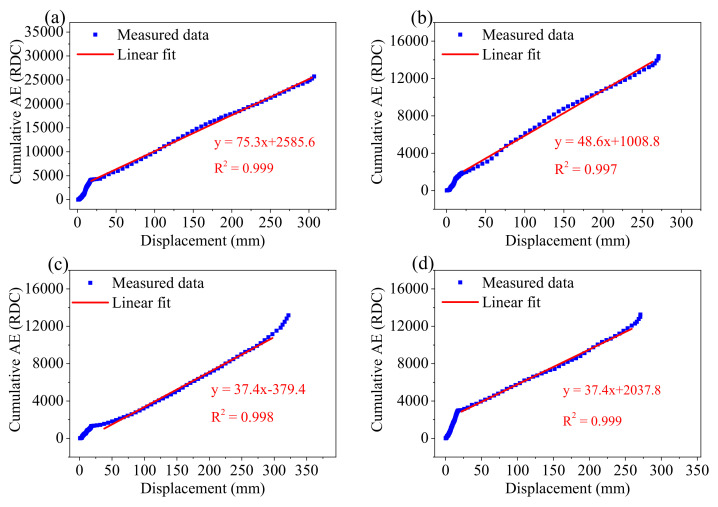
Linear relationship between cumulative ring down count (RDC) and displacement. (**a**–**d**) show the relationship for the four experiments. All data points are the moving average value of 5 s.

Figure 12 shows time series of displacement and cumulative RDC for each experiment. The trend of displacement and cumulative RDC is similar. Both increase slowly in the first two stages, but suddenly increase at about 100 s. There is a satisfactory linear correlation between cumulative RDC and displacement in Figure 13. However, there are local deviations from the linearity at the head and tail segments with different gradients because the slope behaviour transition occurs at a particular time point and, hence, changes both the volume of backfill materials and confining pressures in the backfill. The linear correlation is conducted excluding the data of the two areas where the deviations from the linearity are significant. Figure 13 shows cumulative RDC is linearly proportional to displacement, and R^2^ is close to 1. This conclusion is consistent with previous studies [13,23,25,33]. These four experimental results show that cumulative RDC can quantify displacement at different inclination angles of the container.

### 3.4. Comprehensive Analysis of Kinematics, Dynamics, and AE Measurements

**Figure 14:**
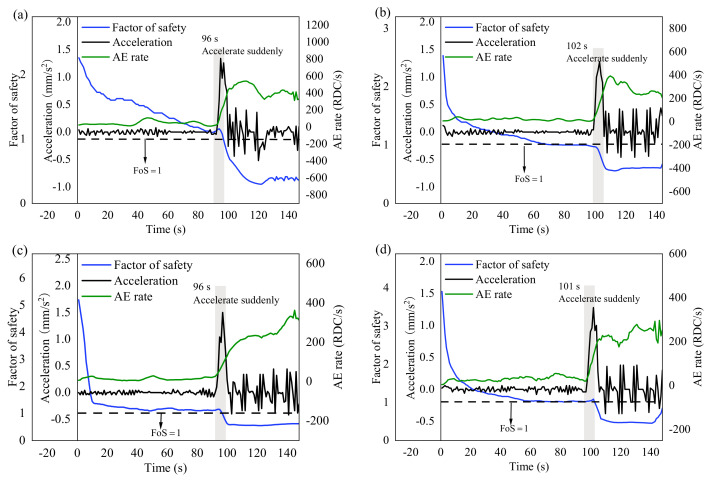
Acceleration, factor of safety (FoS), and ring down count (RDC) plotted against time in (**a**–**d**) for the four experiments. All data points are the moving average value of 5 s.

Acceleration of the sliding body, FoS of slope, and RDC are selected as representative parameters of the kinematics model, dynamics model, and AE monitoring, respectively. Acceleration varies significantly with the three-stage deformation of the landslide. FoS is a typical evaluation index of slope stability with clear physical meaning. RDC is sensitive and reflects internal information of the slope status. Figure 14 shows the time series of acceleration, FoS, and RDC for each experiment. Acceleration and RDC increase sharply at around 100 s, while FoS drops below 1, which indicates the slope enters a dangerous state. Acceleration and RDC have the potential to be effective indications of incipient slope failure.

## 4. Discussion

Dynamics and kinematics models are common methods for slope stability analysis, and AE monitoring is an effective approach to quantify slope deformation behaviour. In this study, the deformation process of the landslide model test was controlled by displacement based on the kinematics model of a three-stage deformation law. Mechanical analysis of the model test was carried out, which was rarely explored in other studies on slope monitoring using AE technology [27,61,68]. The landslide model tests were proved repeatable by three repetitions in each inclined angle, as the same results were produced under the same geometry and material condition.

The landslide experiment is controlled by the displacement. Therefore, the slope stability and landslide evolution are also determined by the displacement. The thrust (Figure 9) and RDC (Figure 13) did not have a consistent variation our a change in the inclination angle, which suggested AE was mainly controlled by the change of strain. AE responded as a function of velocity, and the inclination angle did not seem important for kinematics versus the AE response [61]. Acceleration, FoS, and RDC were selected as representative parameters for comparative analysis. The results showed that acceleration and RDC may be effective indicators for the early warning of progressive landslides. AE is generated by energy dissipation [30]. The quantified relationship between AE and deformation should be explained from the viewpoint of energy dissipation, which is related to the physical essence of AE generation. As the rate of deformation (i.e., velocity) increases, the energy dissipation rate (e.g., AE rate) increases. Therefore, the AE rate is proportional to velocity. The AE rate gradient (i.e., time derivative of the AE rate) is proportional to acceleration (i.e., time derivative of velocity). In terms of Figure 11, Figure 12 and Figure 13, if AE rate is proportional to velocity, cumulative AE certainly should be related to displacement. However, the proportional relationship between deformation and AE partially depend on the settings of the AE system and the properties of an active waveguide [25]. Hence, the coefficient and intercept in the linear equations can be different for each specific case and even dynamic responses of AE appear during the process-based modelling. Other AE parameters like AE hits rate (hits per second), duration, rise time, amplitude, and energy were also examined in parallel with RDC, and RDC was proven to have the best correlation with landslide movement.

Waveform analysis is usually used to understand the physical mechanism of the AE source and focuses on the mechanism of the AE source, the propagation process of acoustic waves, and the response of the transducer. The influencing factors (i.e., the AE source, the propagation process, and the detection response) were uncertain in this study. The AE waveform of the electrical signal was very complicated, and it was very different from the real AE source signal generated by the interaction between the backfill materials and the waveguide. Moreover, due to the large data amount of the waveform file, it was easy to reach the upper limit of the storage capacity. Waveform analysis faced many challenges in terms of collected AE from backfill particles, and it was difficult to obtain more information than parameter analysis. Frequency spectrum analysis was also attempted in this study. AE from an active waveguide are specific to the frequency range of 20–30 kHz [24,25,27,61]. The AE sensor with a dominant frequency range from 15 kHz to 70 kHz was sensitive to and covered a 20–30 kHz frequency band. The monitored range of 20–30 kHz was determined to ensure the results from this study are relevant to field monitoring applications, which use such a frequency range to minimise background noise. As a result of frequency spectrum analysis, the collected AE signal was mainly constrained in the specific range of 20–30 kHz and the change of frequency spectrum was relatively small during the entire three-stage deformation process.

This research focused on exploring the physical process of the model test (e.g., evolution of FoS, force, displacement, and AE). The implications beyond the test for field monitoring required more of an investigation to make it clear. For slope stability analysis, the geotechnical mechanics method is difficult to apply in practice. The commonly used slope FoS is simple with clear physical meaning, but it is not suitable for in-situ monitoring projects because it has enormous difficulties to obtain comprehensive and detailed slope physical and mechanical properties [69]. The AE monitoring instrument is simple and inexpensive with high temporal resolution, real-time usage, on-line access, and is convenient for field application [23]. The judgements of landslide evolution are consistent according to the change patterns of RDC, acceleration, and FoS, which suggest that AE and acceleration may be effective indicators for landslide monitoring and early warning. Inverse velocity in Figure 8 seems to be another representative parameter for the kinematic model, and some studies explored this further [70,71]. Continuous functioning of the AE rate (RDC per second) was also considered as an alternative approach because it can provide a more informative representation of the AE activity within a given time period [72].

The landslide model built in this study provides an example for physical analysis. However, differences exist in the temporal and spatial scale between the model test and the actual landslide. The failure surface in the model test was restrained along the flat plane parallel to the loading direction. However, it is more likely to fail along a circular surface for a natural soil slope. Considering spatial variation and strain hardening/softening, which changes with the development of failure surface, the relationship between FoS and AE behaviour in this model test is different from that in the field. It is difficult to set an early warning threshold for acceleration and RDC based on the model test. Some problems need to be explored to apply the knowledge from the model test to field monitoring. Due to the depth of waveguide sticking into the artificial substratum being 6 cm, the waveguide and both sides of the test container hindered the slope mass during the sliding process. These hindrances mobilise as a function of the applied force (thrust) and vary with time. It is challenging to directly determine the hindrances. Therefore, in the mechanical analysis, the average of the other resistance f was used as a constant value on behalf of these hindrances, which is not consistent with the actual situation. In fact, the mobilised friction angle assumed as a constant value changed with increments of shear strain.

## 5. Conclusions

This study focused on the correlation between AE monitoring and a dynamics model of the soil landslide using a model test and physical analysis. AE technology, displacement, and mechanical measurement were applied to monitor the landslide experiment. Displacement, velocity, acceleration, thrust, and RDC were obtained for comparative investigation, and these parameters were analysed to discover the variation law and mutual relationship. The primary conclusions of this study are as follows.

(1) A concise calculation equation of FoS was proposed for the model test. The quantitative relationship was established between FoS and thrust, and the linear relationship was found between the reciprocal of FoS and the thrust.

(2) The trend of FoS, displacement, velocity, acceleration, thrust, and RDC was consistent in the three-stage deformation process of the soil landslide. The linear relationship between cumulative RDC and displacement suggested RDC was useful to quantify the progressive deformation of the soil slope.

(3) The change of FoS, RDC, and acceleration was synchronous for the displacement dependent landslide process. Acceleration and RDC increased and FoS dropped below 1 at the beginning of the accelerating stage, which indicated acceleration and RDC may be useful for the landslide’s early warning.

## Figures and Tables

**Table 1:** Properties of the soil sliding body.

Length (cm)	Width (cm)	Height (cm)	Bulk Density (Mg·m^−3^)	Internal Friction Angle φ (°)
70	34	30	1.6	24

**Table 2:** Details of the four experiments.

General Conditions	Test Number	Inclination Angle	Velocity Control
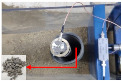 Waveguide (Copper pipe): Length of 1 m External diameter of 30 mm Internal diameter of 20 mm Silica sand particles: Average size of 6 mm Rubber inclinometer: External diameter of 60 mm Internal diameter of 55 mm	(a)	5°	Stage one ${v\;=\;t}^{-0.5}\;\mathrm{mm}/s$ (1) t∈(0,50) s
	(b)	10°	Stage two v = 0.2 mm/s (2) t∈(50,100) s
	(c)	15°	Stage three $v\;=\;\frac{5^{t-100}}{500}\;\mathrm{mm}/s$ (3) t∈(100,105)
	(d)	20°	$v_{max}\;=\;6.25\;\mathrm{mm}/s$ t∈(105,150) s

**Table 3:** Calculated *a* and *b* under different inclination angles of the container.

Angle θ (°)	5	10	15	20
*a* (kN)	1.0387	1.0358	1.0311	1.0244
*b* (kN)	0.0498	0.0992	0.1478	0.1954

## Data Availability

Data sharing not applicable.
